# All-Soft Skin-Like Structures for Robotic Locomotion and Transportation

**DOI:** 10.1089/soro.2019.0059

**Published:** 2020-06-02

**Authors:** Jianglong Guo, Chaoqun Xiang, Andrew Conn, Jonathan Rossiter

**Affiliations:** SoftLab, Bristol Robotics Laboratory, University of Bristol, Bristol, United Kingdom.

**Keywords:** active functional skins, dielectric elastomer actuators, soft electroadhesives, soft-smart composites, soft-smart robots

## Abstract

Human skins are active, smart, and stretchable. Artificial skins that can replicate these properties are promising materials and technologies that will enable lightweight, cost-effective, portable, and deployable soft devices and robots. We show an active, stretchable, and portable artificial skin (ElectroSkin) that combines dielectric elastomer actuators (DEAs) and soft electroadhesives (EAs) in a fully compliant multilayer composite skin-like structure. By taking advantage of the common characteristics of DEA and EA, we define regions of the composite artificial skin as either active or passive. Active areas can be exploited as electromechanical actuators or as electrostatic gripper elements, or both simultaneously. This embedded multimodality delivers a new technology of deformable active skins that can grip and move objects and self-locomote. ElectroSkins can be fabricated using all-soft elastomers and readily available conductive materials. We demonstrate their capabilities in the first soft self-actuating conveyor belt, with a conveyoring speed of 0.28 mm/s, and a pocketable fully soft crawler robot. This new, self-actuating, self-gripping, and self-locomoting soft artificial skin has the potential to significantly impact on functional soft-smart composites, deployable robots, soft-smart conveyoring, and compliant gripping and manipulation applications.

## Introduction

Pocketable and deployable devices, fabricated from intrinsically robust and compliant soft-smart materials and structures, that can self-locomote and move objects will deliver important new capabilities. These range from fast deploying rescue and space robots to self-adapting grippers and morphologically adapting robots. Current soft functional devices are limited, for example, by the need for frames^1,2^ upon which to mount actuators or the use of fluidic drive mechanisms.^3,4^ The need is, therefore, for devices with completely soft bodies and that are driven by readily controllable and easily stored electric energy. Although prior research has shown electrically driven and active soft devices using shape memory alloys,^5–7^ pneumatic actuators,^8,9^ and electric motors,^10^ they are energy inefficient, rate-limited, or introduce structurally complex and bulky components in the use of pumps or motors.

**FIG. 1.**
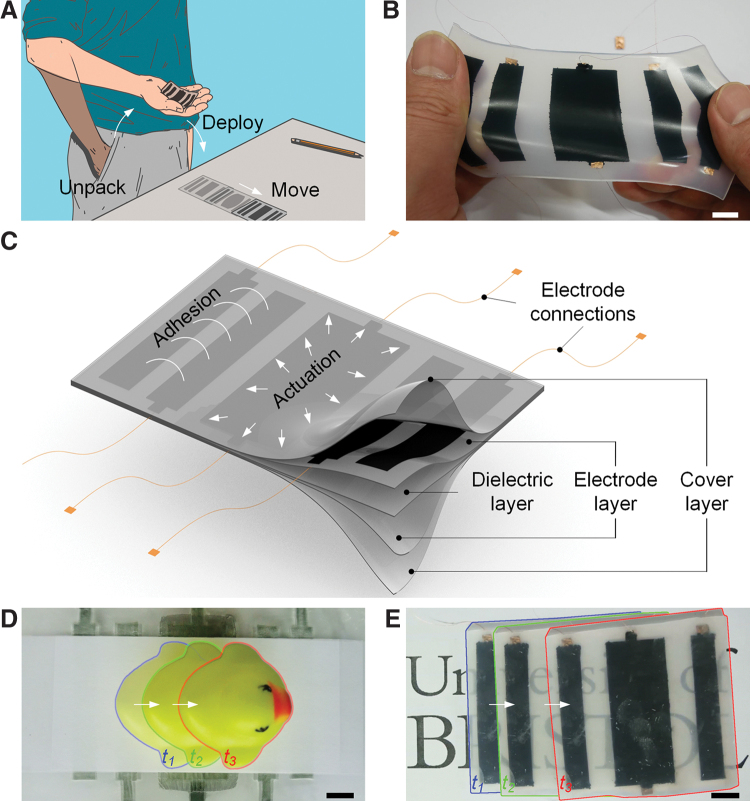
Active soft-smart structures with self-actuating, self-gripping, and self-locomoting capabilities. **(A)** Pocketable and deployable fully soft crawling robot concept. **(B)** A fully soft ElectroSkin robot stretching in hand. **(C)** Schematic diagram of the fundamental ElectroSkin design showing regions powered for electroadhesion and actuation. **(D)** An ElectroSkin conveyor moving a *yellow duck* on a piece of office paper. **(E)** A fully soft ElectroSkin robot self-locomoting across a surface. Scale bars denote 1 cm. Color images are available online.

To overcome these limitations, we present an approach to all-soft robots by exploiting the common characteristics and materials of two emerging actuation technologies. Figure 1A shows the concept of a highly deformable fully soft skin-like robot that can be pulled out of a pocket in its compressed (rolled or scrunched up) form and thrown onto a surface. It then moves autonomously or by remote control. In this study we define a skin-like robot as a structure/device whose thickness is at least one order magnitude smaller than its width/length. We exploit the fusion of two complementary electrically driven technologies for active and electrically controllable actuation, adhesion, and gripping for deployable soft functional devices: dielectric elastomer actuators (DEAs), made of deformable dielectric membranes sandwiched between two compliant electrodes, and soft-stretchable electroadhesives (EAs), comprising compliant planar electrodes attached to, or embedded in, a soft dielectric.

DEAs are essentially soft and variable parallel capacitors that can be deformed under the application of an electric field due to Maxwell pressure, thus generating significant stresses and strains. When an electric field (usually in the range of MV/m) is applied, induced Maxwell pressure between the two electrodes deforms the structure and the effective Maxwell stress, $\sigma_{M}$, compressing the dielectric membrane is usually characterised as^11^

(1) $$\sigma_{M}\propto \varepsilon_{0}\varepsilon_{r}E^{2},$$

where $\varepsilon_{0}$ is the permittivity of the free space, $\varepsilon_{r}$ is the relative permittivity of the dielectric, and *E* is the electric field. This electrically induced mechanical deformation is captured in the structure to deliver useful actuation such as artificial muscle contraction,^12–14^ multidegree-of-freedom actuation,^15^ pumps,^16^ and adjustable lenses.^17^ DEAs are considered as promising artificial muscles because they are lightweight and cost-effective, can generate significant strains >500%,^18^ show fast response,^19^ and have high energy densities.^11^ DEAs can also be produced by flexible materials and can be comfortably fused with other materials such as fluids^20,21^ and magnets.^22^

Soft EAs are predominately variable coplanar capacitors that can be used to produce controllable adhesion under the application of a voltage. Electroadhesion employs electric fields to generate an electrically controllable adhesive force between the EA device and the object. The application of an electric field (also in the range of MV/m) between the electrodes causes polarization in the touched object and induced electrostatic attraction forces between the EA device and the object, and the EA force generated on insulating materials, $F_{EA}$, is usually characterized as (two-dimensional [2D] representation)^23,24^

(2) $$F_{EA}\propto PE,$$

where *P* is the polarization between the EA pad and the substrate material and can be denoted by $\varepsilon_{0}\left(\varepsilon_{r}-1\right)E$ for homogeneous, linear, and isotropic dielectric materials. Typically, this force is employed in a holding, active adhesion, or gripping mechanism.^25–31^ Most EAs employ rigid or flexible substrates but some stretchable soft EAs have been fabricated.^28–34^ EA is a promising controllable adhesion and material handling technology because it has reduced complexity in structure and control, low energy consumption, increased adaptability to various surfaces, and can handle gentle and flexible objects that are a challenge to conventional mechanical grippers.^28–30^

**FIG. 2.**
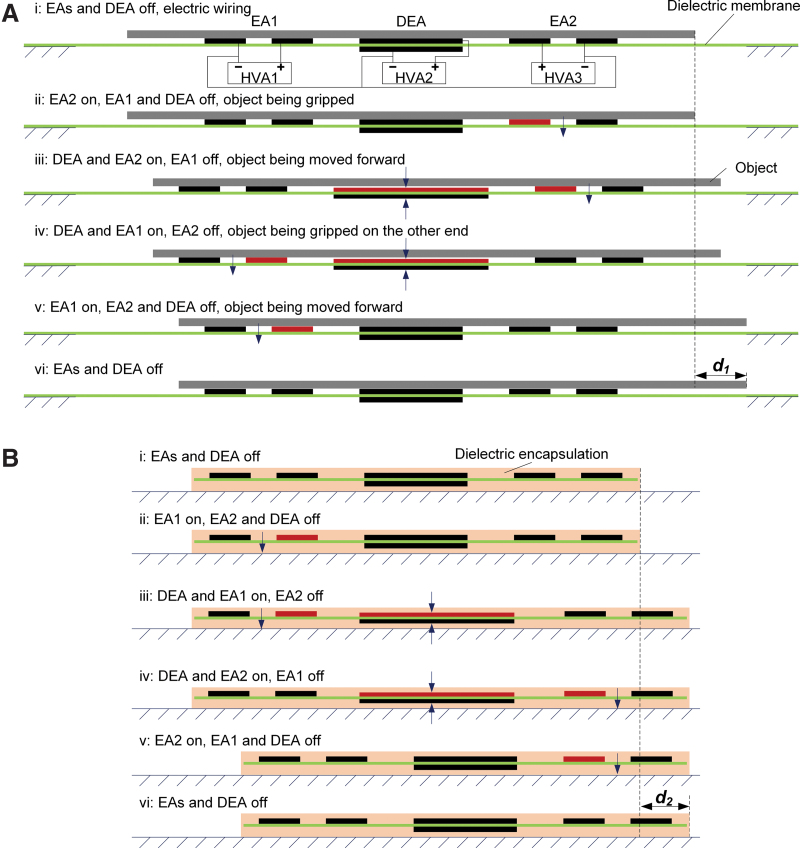
ElectroSkin operation principles. **(A)** Schematic cross-sectional view of the basic operation procedure of the 6:5 soft ElectroSkin conveyor for moving objects forward. Three HVAs are used. All three negative terminals are connected to ground. The operation procedure contains six steps: (i) put the object on the conveyor belt when no voltage is applied; (ii) turn on the EA2; (iii) turn on the DEA; (iv) turn off the EA2 and turn on the EA1; (v) turn off the DEA; (vi) turn off the EA1 and start the next cycle if needed. For moving objects backward, EA1 should be turned on first rather than EA2. **(B)** Schematic cross-sectional view of an encapsulated 6:5 entirely soft ElectroSkin crawler robot. The basic operation procedure for crawling forward contains six steps, as shown. The electric connections for the soft ElectroSkin robot are the same as the ElectroSkin conveyor. DEA, dielectric elastomer actuator; EA, electroadhesive; HVAs, high-voltage amplifiers. Color images are available online.

DEAs and soft EAs have previously been used through integrated configurations^29,30^ or modular compositions^1,2,35,36^ as gripping, actuating, adhesive, and locomoting structures and devices. In this study we exploit the similarities in morphology and transduction mechanisms of DEAs and EAs to develop a fully soft monolithic skin-like composite structure—ElectroSkin—that shows simultaneous or separate actuation and gripping (Fig. 1B, C). By taking advantage of the common and contrasting characteristics of DEA and EA electrodes, we define regions of the ElectroSkin as either active or passive. Active areas can be exploited as electromechanical actuators or as EA gripper elements, or both simultaneously. This embedded multimodality delivers a new technology of deformable active skins that can employ self-adhesion to attach to and grip surfaces and objects, or employ self-adhesion and self-actuation to locomote and move objects. We describe the design and fabrication of ElectroSkin and characterize its basic operation in both DEA actuator and EA adhesive roles. We demonstrate the potential of ElectroSkin in two example applications: (1) a monolithic, lightweight, noise-free, soft, and low energy consumption self-actuating conveyor, as presented in Figure 1D, and (2) a monolithic, lightweight, easy-to-fabricate, and pocketable fully soft self-locomoting crawler robot, as presented in Figure 1E. These show the great potential of ElectroSkin that can help develop self-actuating, self-gripping, and self-locomoting functionalities in future soft robots.

## ElectroSkin Design and Operation Principles

ElectroSkin is fully soft and stretchable, as demonstrated in Figure 1B, and comprises three basic elements: (1) a compliant dielectric middle layer, (2) two or more compliant electrodes, arranged in-plane on one side of the dielectric or parallel on opposite sides, and (3) an optional encapsulating layer. The electrodes can be configured in any patterns and electrically controlled so that any area of the dielectric can be turned into a DEA actuator (by energizing a pair of parallel through-plane electrodes) or an EA gripper (by energizing two in-plane electrodes) or both (by combining in-plane and through-plane electrodes). This flexibility provides the basic framework for arbitrarily complex actuation and manipulation tasks. One embodiment as a soft self-actuating and self-gripping conveyor skin is shown in Figure 1D. This conveyor is configured with a total of six electrodes, comprising a central parallel DEA pair and two lateral pairs of coplanar electrodes performing EA gripper functions. We define the electrode layout as having a six-electrode five-unit configuration (6:5 for short), where a unit is a discrete electrode area (single or double sided) that is in-plane separable from other electrode areas. Here no encapsulation need be employed since the conveyor was mounted on a plastic frame. Activation of the central DEA causes the two EA pairs to move apart in the plane of the material. Activation of each of the EA electrode pairs generates local EA forces that can grip an object placed on the conveyor. By controlling the timing of activation of the DEA and EA pairs, we implement a grip-move-release-relax cycle that incrementally moves an object across the surface of the conveyor. The full one operation cycle of a 6:5 ElectroSkin soft conveyor is shown in Figure 2A, at the end of which a flat gray object is shown to move laterally a distance of *d_1_*. The cycle is defined by six steps: (i) electric wires are connected to high-voltage amplifiers (HVAs); (ii) the right EA unit is turned on to grip the object; (iii) the DEA unit is turned on to move the object to the right; (iv) the right EA unit is turned off and the left EA unit is turned on, to maintain the position of the object on the conveyor; (v) the DEA is turned off, further moving the object to the right; and (vi) finally, the second EA unit is turned off and the next cycle can be started (Supplementary Movies S1, S2, S3). To move the object in the opposite direction, activation of EA1 and EA2 is reversed in the cycle. The duration of each cycle can be varied and tailored to specific applications.

By simply encapsulating the 6:5 ElectroSkin on the top and bottom with a thin insulating elastomer layer and releasing the skin from the frame, we can generate a self-actuating and self-gripping crawling robot. The encapsulating elastomer is sufficiently stiff to capture the prestrain of the dielectric layer while being soft enough for the skin to be easily stretched, folded, and deformed. The resulting robot can be rolled up and put in one's pocket, then subsequently pulled out, placed on a surface, and it will move across the surface (electrical power in our demonstrations is provided externally through thin wired connections). Figure 2B shows the schematic of the resulting crawling robot and the actuation pattern that causes the robot to locomote across a surface. In its simplest form, this actuation pattern can be the same as that for the conveyor but reversed since the robot itself is moving to the right, rather than the object as in the case of the conveyor. After one full actuation cycle, the robot has moved a distance of *d_2_* to the right, ready for the next cycle.

## Experimental Section

### ElectroSkin fabrication materials and procedure

ElectroSkins can be fabricated using all-soft elastomers and readily available conductive materials. The fabrication procedures comprise the five steps below (with dimensions given for the 6:5 design). The first three steps alone can be used to make the soft ElectroSkin conveyor belt. Fabrication of soft ElectroSkin crawler robots requires all steps 1–5. Step 1: Prestretch the VHB film on the laser-cut acrylic plate. A 1 mm thick VHB 4910 dielectric membrane (3M) was prestretched by a biaxial stretcher from diameter 40 to 190 mm, yielding a linear strain of 475%, area strain of 2256%, and final membrane thickness of 44 μm. A 5 mm thick acrylic plate was laser cut into a rectangular frame with inner dimensions of 85 × 130 mm and outer dimensions of 95 × 140 mm. Step 2: Cut the masks and bond them onto the VHB film. A 65 μm thick Q-connect A4 punched pocket (Interaction-Connect, Belgium) was used as the masks for casting the conductive electrodes on the VHB film. The masks were cut by a Cricut 2D computer-controlled material cutter (Provo Craft & Novelty, Inc.). Step 3: Mask print the conductive silicone electrodes and wire the electrical connections. In this study, we fabricated the curable electrodes by mixing 20% wt conductive carbon grease (MG Chemicals, Canada) with Ecoflex 00–10 (Smooth-On, Inc.) silicone elastomer. The resulting electrode material was conductive, stretchable, cost-effective, and fused well with a subsequent encapsulating layer. The electrodes were mask printed onto the VHB membrane, and the masks were then removed. The electrical connections were completed (copper tape was applied before curing to ensure a good electrical connection), and the ElectroSkin was placed in an oven to cure at 50°C for 4 h. The top side of the ElectroSkin was then lightly brushed with talcum powder to remove any intrinsic adhesion before testing. Step 4: Optionally encapsulate the conveyor. To make an entirely soft ElectroSkin crawler robot, a 1 mm thick layer of Ecoflex 00–30 was cast onto both sides of the ElectroSkin. The Ecoflex encapsulant was cured at room temperature for 8 h (4 h on each side). Step 5: Release the soft robot from the acrylic frame. After the encapsulant was cured, the rigid acrylic frame was removed. The entirely soft ElectroSkin robot was then placed on a clean and flat acrylic plate, to which it adhered due to Ecoflex's intrinsic adhesion. The edges of the robot were then sealed with a bead of Ecoflex 00–30. After curing for another 4 h, the soft ElectroSkin robot was peeled off the acrylic plate and trimmed to a neat shape. One side of the robot was dusted with talcum power to remove the intrinsic adhesion before testing.

### ElectroSkin conveyor belt and crawler robot displacement measurement and demonstration

Three 10 kV ultravolt HVAs (10HVA24-BP1; Advanced Energy Industries, Inc.) were used to energize the EA and DEA actuators within the ElectroSkin. A laser displacement sensor (LK-G3001; Keyence) was used to record the displacement of a yellow toy duck (6.1 g) bonded to a piece of paper (44 × 118 × 0.1 mm) on the ElectroSkin conveyor belt. A 50 frame per second Panasonic DMC-G80 camera (Panasonic, United Kingdom) was used to record the dynamic area change (see Supplementary Fig. S1) of the DEAs, and the movement of ElectroSkin conveyor belts and crawlers.

## Results

### ElectroSkin design theoretical considerations and empirical geometric parameter selection

#### ElectroSkin conveyor force analysis

**FIG. 3.**
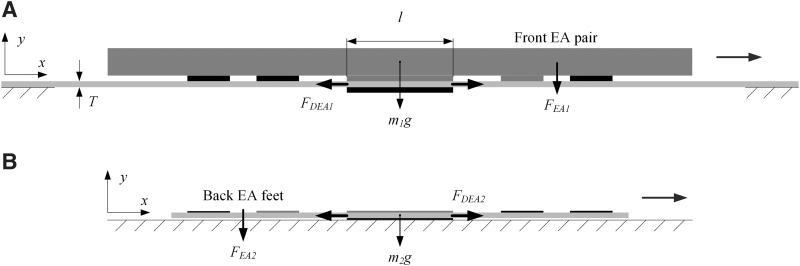
Force analysis of the 6:5 ElectroSkin design. **(A)** Forces exerted on the 6:5 ElectroSkin conveyor belt. **(B)** Forces exerted on the 6:5 ElectroSkin crawler.

After putting an object on top of the soft ElectroSkin conveyor, we first energize the front EA pair, as shown in Figure 3A, to grip the object using the normal EA force, $F_{EA1}$. We then energize the central DEA and generate the in-plane DEA force, $F_{DEA1}$. This is employed to move the object, and should be greater than the friction force between the conveyor and the object. Also, the tangential EA force holding the object at the front EA pair should be greater than the friction force between the soft conveyor and the object. Neglecting other surface forces such as the Van der Waals force, we have

(3) $$\left\{\begin{array}{c} F_{DEA1}>\mu_{1}m_{1}g\\ F_{EA1}>m_{1}g \end{array}\right.,$$

where $\mu_{1}$ is the static frictional coefficient between the object and the ElectroSkin conveyor, $F_{EA1}$ is the normal EA force between the front EA electrodes and the object, and *m*_1_ is the mass of the object.

The total input energy of the 6:5 DEA-EA ElectroSkin design is

(4) $$W=0.5\left[C_{DEA}\left(t\right)\Phi_{DEA}^{2}\left(t\right)+C_{EA1}\left(t\right)\Phi_{EA1}^{2}\left(t\right)+C_{EA2}\left(t\right)\Phi_{EA2}^{2}\left(t\right)\right],$$

where $C_{DEA}$ is the time varying capacitance of the DEA, $\Phi_{DEA}$ is the applied voltage to the DEA electrodes, $C_{EAi}$ is the time varying capacitance of the EA unit, $\Phi_{EAi}$ is the applied voltage to the EA electrodes, and *i* = 1, 2 denotes the front and back EA unit. This energy is dissipated through moving objects (conversion into potential energy and friction losses) and through DEA/EA actuation (material viscoelastic losses, DEA/EA dielectric loss and leakage current). For one cycle, to move a mass *m*_1_ a distance *d*_1_, we should have

(5) $$W>\mu_{1}m_{1}gd_{1}.$$

Therefore, the heavier the object mass, the higher the power consumption needed, requiring the application of greater voltages. These considerations and the fundamental DEA Equation (1) suggest that ElectroSkin materials must have the following characteristics: (1) Encapsulating materials should be compliant enough to permit a sufficient DEA strain but stiff enough to maintain a sufficient prestretch of the dielectric membrane; (2) DEA membranes should have high dielectric constants to effectively transduce electrical energy into Maxwell stresses; and (3) Both encapsulating and DEA materials should have low viscoelasticity to reduce losses, toward the ideal case, where $W=\mu_{1}m_{1}gd_{1}$.

#### ElectroSkin crawler robot force analysis

After putting the soft ElectroSkin crawler robot on a substrate, we first energize the back EA feet, as shown in Figure 3B, to hold the robot using the normal EA force, $F_{EA2}$. We then energize the central DEA and apply the resulted in-plane DEA force, $F_{DEA2}$, to locomote the front EA feet. To successfully locomote the front EA feet forward, the in-plane DEA force and the tangential EA force should both be greater than the friction force between the front EA feet and the substrate. We then have

(6) $$\left\{\begin{array}{c} F_{DEA2}>\mu_{2}m_{2}g\\ F_{EA2}>m_{2}g \end{array}\right.,$$

where $\mu_{2}$ is the static frictional coefficient between the substrate and the ElectroSkin crawler, $F_{EA2}$ is the normal EA force between the back EA feet and the substrate, and *m*_2_ is the mass of the ElectroSkin crawler. For silicone surfaces, the static frictional coefficient is usually significantly >1, resulting in a tangential EA force greater than the normal EA force. Typically, the mass of the crawler robot, *m*_2_, is much less than the mass of the object moved by the conveyor, *m*_1_. Therefore, in practice, $F_{DEA2}$ can be much smaller than $F_{DEA1}$. This means that a lower^37^ operating voltage could be used for the crawler robot. As can be seen from Equations (3) to (6), the in-plane DEA force of the ElectroSkin design should be greater than the friction force between the ElectroSkin and the conveyored object or the crawling surface. Using the Gent model for a state of equibiaxial strain,^38^ the in-plane DEA force can be expressed as

(7) $$F_{DEA}=lT\left[\frac{G\left(\lambda -\lambda^{-5}\right)}{1-\frac{2\lambda^{2}-\lambda^{-4}-3}{J_{\lim}}}-\varepsilon_{0}\varepsilon_{r}\lambda^{3}{\left(\frac{\Phi }{T}\right)}^{2}\right]>\mu_{1}m_{1}g,$$

where *l* is the nominal DEA electrode width, *T* is the nominal dielectric membrane thickness, *G* is the shear modulus of the dielectric membrane, Φ is the applied voltage, $J_{\lim}$ is the material constant related to the limiting stretch, and λ is the in-plane stretch.

The EA force exerted on a material has been obtained by using the Maxwell stress tensor method,^39,40^ and the electrostatic (neglecting the effects of magnetism) Maxwell stress tensor, $T_{ij}$, is defined, in component form, as

(8) $$T_{ij}=\varepsilon \left(E_{i}E_{j}-\frac{1}{2}\delta_{ij}E^{2}\right),$$

where ε is the dielectric permittivity, $\delta_{ij}$ is the Kronecker delta, and the electric field *E* is represented by −∇Φ, where the electric potential, Φ, in a dielectric medium, satisfies the Laplace equation, $\nabla^{2}\Phi =0$.

The EA force acting on a substrate of a unit length can then be calculated as (in 2D representation)

(9) $$\begin{array}{cc} F_{EA} & =\oint_{S}T_{yy}dS\\ & =\frac{1}{2}\varepsilon_{0}\int_{0}^{2w+s}\left[E_{y}^{2}\left(x,y,t\right)-E_{x}^{2}\left(x,y,t\right)\right]dx>m_{2}g \end{array},$$

where *w* is the EA electrode width, *s* is the space between the two EA electrodes, *E_x_* and *E_y_* are the electric field components in the air gap between the EA device and the substrate material. In our experiments, the encapsulating material (Ecoflex 00–30) is different to dielectric elastomer membrane material (very high bond [VHB] 4910) and, therefore, the dielectric constants in Equations (1) and (2) will differ. In practice, we may choose to use the same material for both, further simplifying fabrication and analysis.

#### ElectroSkin empirical geometric parameter selection

**FIG. 4.**
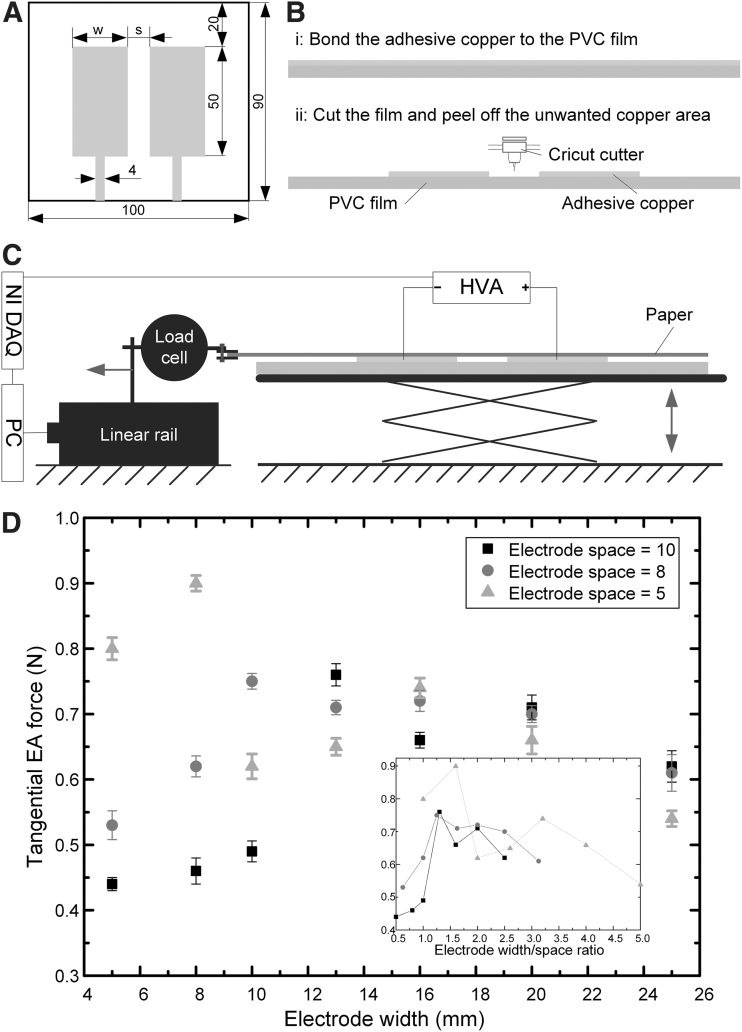
Two-electrode EA geometric optimization. **(A)** The two-electrode EA electrode geometric design. **(B)** The EA fabrication procedure: (i) bond the adhesive copper to the PVC film; (ii) cricut the copper laminate and peel off the unwanted copper area. **(C)** The schematic diagram of the tangential EA force test rig. **(D)** The tangential EA forces for electrode spaces of 5, 8, and 10 mm when the electrode widths were set as 5, 8, 10, 13, 16, 20, and 25 mm. The error bars denote 1 standard deviation of the five tests of each EA design. The *inset* shows the relationship between electrode width/space ratios and tangential EA forces on a sheet of paper for the two-electrode EA design. PVC, polyvinyl chloride.

Empirical EA electrode geometric optimization was performed here because current EA theoretical and simulation models fail to predict both the normal and tangential EA forces and it is impractical to include surface texture, environmental conditions, and dynamic dielectric properties under high electric fields into EA models, although these factors will influence the EA force.^23–28^ The geometric optimization of the EA pair unit was based on an experimental study based on a customized and repeatable EA electrode design, fabrication, and adhesive force test platform, as presented in Figure 4. The EA electrode width and electrode space were set as 8 and 5 mm, respectively, based on the results shown in Figure 4. The width of the DEA electrodes was set as 20 mm, which was sufficient for demonstration of conveyoring actions.

To select the geometric parameters of the EA unit to achieve the maximum shear EA adhesive force, a customized and repeatable EA electrode design, fabrication, and adhesive force test platform has been established, as shown in Figure 4. The EA electrode geometric design is shown in Figure 4, where the overall dimension of the EA was set as 90 × 100 mm. The electrode length was set as 50 mm. The electrode space was set as 5, 8, and 10 mm. For each electrode space, seven different electrode widths were designed: 5, 8, 10, 13, 16, 20, and 25 mm. The EA fabrication procedure is shown in Figure 4B, containing mainly two steps. First, a 40 μm thick A4 size adhesive copper tape (Cat Music, United Kingdom) was bonded to a 0.3 mm thick A4 size clear polyvinyl chloride (PVC) sheet (Binding Store Ltd., United Kingdom). Second, the copper laminate was cut by the Cricut cutter based on the designated geometric parameters. The unwanted copper area was then peeled off from the PVC sheet, leaving the EA design shown in Figure 4A for force testing. A customized test rig was built to measure the tangential EA force. The schematic diagram of the force test rig is presented in Figure 4C. An inline miniature S-Beam load cell (Applied Measurements Ltd., United Kingdom) was used to measure the adhesive force. A Zaber linear rail (X-LSQ150B-E01; Zaber Technologies, Inc.) was used to pull the paper away from the EA after charging for 60 s using a 5 kV ultravolt high-voltage power supply (5HVA24-BP; Advanced Energy Industries, Inc.). Five kilovolts were applied to the EA pads. The movement speed was 50 mm/s. An NI USB-6343 X Series DAQ device (National Instruments, United Kingdom) was used to record the adhesive forces and control the output voltage of the HVA. During the force measurement, five tests were conducted for each EA pad. In addition, 2 s discharging by the HVA plus 5 min waiting time was employed between tests. All the tests were conducted when the temperature was 21.7°C ± 0.1°C and relative humidity was 31% ± 1%. The tangential EA forces for electrode spaces of 5, 8, and 10 mm when the electrode widths were set as 5, 8, 10, 13, 16, 20, and 25 mm are presented in Figure 4D. For each electrode space, there is an optimum electrode width. For electrode spacings of 5, 8, and 10 mm, the optimum electrode widths were 8, 10, and 13 mm, respectively. The maximum tangential EA force was achieved when the electrode space and width were 5 and 8 mm, respectively. As shown in the inset in Figure 4D, it is clear that we should choose electrode width/space ratio between 1.3 and 1.6 for a two-electrode EA pad design to achieve the maximum adhesive forces on papers. EA electrode width of 8 mm and electrode space of 5 mm were thus used for the 6:5 ElectroSkin design. Empirical optimization was performed because current EA theoretical and simulation models fail to accurately predict both the normal and tangential EA forces and it is impractical to include surface texture, environmental conditions, and dynamic dielectric properties under high electric fields into EA models, although these factors will influence the EA force.

**FIG. 5.**
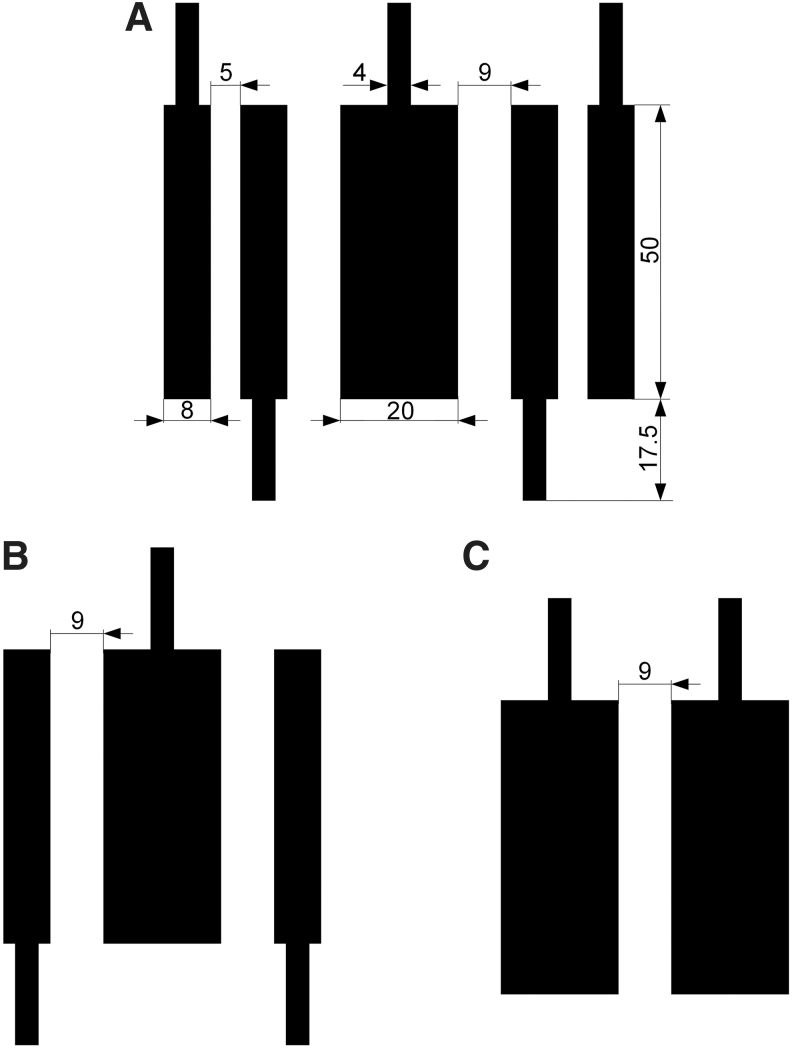
ElectroSkin conveyor belt electrode geometric designs. **(A)** Dimensions of the 6:5 design. The unit for the dimensions is millimeter. **(B)** The 4:3 design. **(C)** The 4:2 design.

The electrode geometric dimensions of the 6:5 ElectroSkin design are shown in Figure 5A. The space between the DEA and EA electrode was set as 9 mm. The electrode width and length for the electric connections were set as 4 and 17.5 mm. For the 4:3 (four-electrode, three-unit) ElectroSkin conveyor belt design, only the middle three electrode units of the 5-unit design was used (Fig. 5B). For the 4:2 (4-electrode, 2-unit) ElectroSkin conveyor belt design, two DEAs with the same electrode width and length were used (Fig. 5C). The space between the two DEAs was 9 mm. The width of the DEA electrodes was set as 20 mm, which was sufficient for demonstration of conveyoring actions. The operation principles of the 4:3 (Supplementary Fig. S3A) and 4:2 (Supplementary Fig. S3B) designs are presented in Section 2 in the Supplementary Data.

### Control strategies and displacements of soft ElectroSkin conveyors

**FIG. 6.**
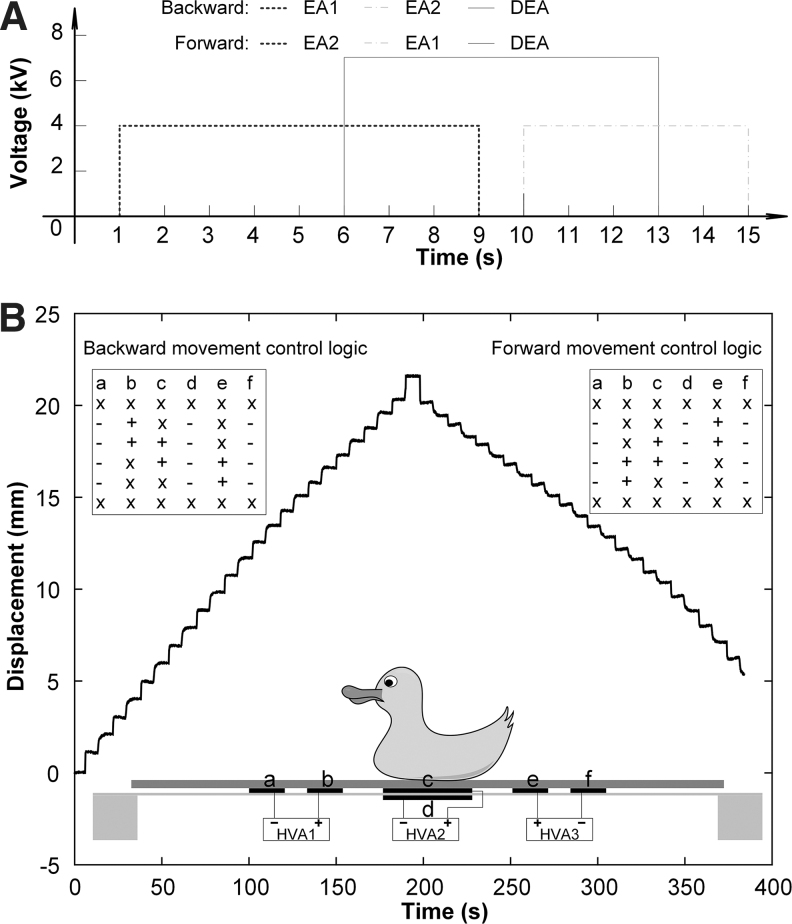
Voltage application strategy and displacement of a soft 6:5 ElectroSkin conveyor belt. **(A)** Voltage application strategy for the 6:5 ElectroSkin conveyor belt. **(B)** Backward and forward displacement curve of a *yellow toy duck* bonded to a paper sheet (Supplementary Movie S1) on the ElectroSkin conveyor belt for a duration of 12 cycles. *Inset* tables show activation logic where “x” denotes that no voltage is applied to the electrodes; “+” denotes the positive high voltages; and “−“ denotes the ground.

Three HVAs (10HVA24-BP1, Advanced Energy Industries, Inc.) were used to control the voltage application to the EA and DEA electrodes within the ElectroSkin. A laser displacement sensor (LK-G3001; Keyence) was used to record the displacement of a yellow toy duck bonded to a piece of paper (44 × 118 × 0.1 mm) on the ElectroSkin conveyor belt. The voltage control strategy is shown in Figure 6A, where 4 kV is applied to the EA pairs and 7 kV is applied to the DEA. In this study, we define the duration of each cycle as 16 s (as shown in Fig. 6A). Figure 6B shows the basic forward and backward movement control logics and the resulting displacement of the piece of paper supporting the yellow toy duck (6.1 grams; Supplementary Movies S1, S2, S3) against time. The difference in forward and backward velocities is attributed to the slight variation in friction coefficient across the skin and differences in exerted EA adhesive force due to fabrication tolerances. The conveyor test was repeated three times. The yellow duck moved 21.57 ± 0.031 mm in 12 cycles, as presented in Figure 6B.

**FIG. 7.**
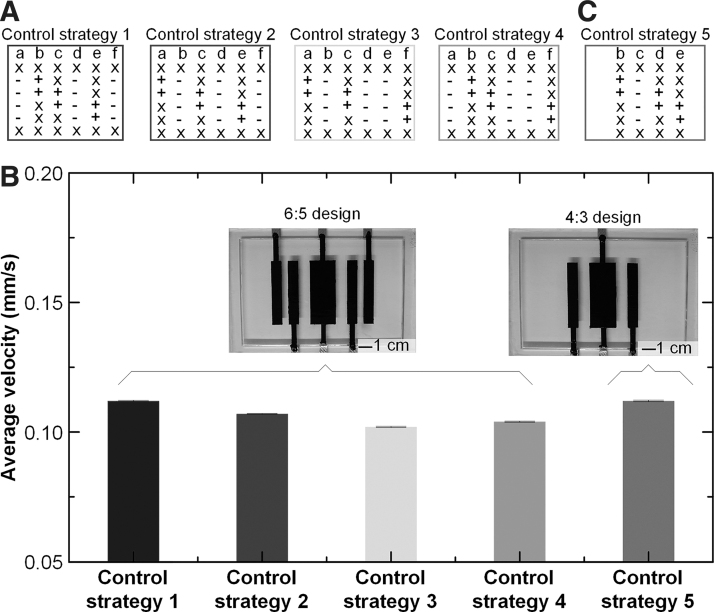
Control logics and average velocities of soft ElectroSkin conveyor belts. **(A)** Different control logics for the 6:5 ElectroSkin conveyor. **(B)** Average conveyoring velocities based on the control strategies shown in Supplementary Figure S5A. The error bars denote 1 standard deviation of the three tests for each control strategy. **(C)** Control logic for the 4:3 ElectroSkin conveyor.

If we define stroke as the displacement per body length per cycle, the average stroke of this ElectroSkin conveyor belt is 1.38%. Various other control strategies can be used to move materials based on the design shown in Figure 6B. If we fix the voltage application to the DEA, there are four different control logics, as shown in Figure 7A. The displacements of the yellow duck based on the other three strategies were 20.52 ± 0.031 mm (control strategy 2), 19.48 ± 0.042 mm (control strategy 3), and 19.98 ± 0.036 mm (control strategy 4) in 12 cycles. The average strokes of the three strategies were, therefore, 1.32%, 1.28%, and 1.25% for control strategies 2, 3, and 4, respectively. Owing to the EA interaction between the electrode b/e and c, the displacements based on control strategies 2, 3, and 4 were slightly smaller than the displacement based on control strategy 1. The conveyoring speeds based on control strategies 1, 2, 3, and 4 were 0.112 ± 0.00016, 0.107 ± 0.00016, 0.104 ± 0.00019, and 0.102 ± 0.00022 mm/s, respectively, as shown in Figure 7B. We can take advantage of the EA interactions between electrodes b/e and c to simplify the 6:5 design. A four-electrode design, rather than the six-electrode design shown in Figures 1 and 2, can also be used to move materials. A four-electrode three-unit (4:3) ElectroSkin actuator design, shown in inset in Figure 7B, can be fabricated by simply removing the outer two electrodes of the 6:5 design. To convey materials, the simplified control strategy 5, as presented in Figure 7C, is used. The 4:3 ElectroSkin conveyor belt moved the yellow toy duck 21.54 ± 0.051 mm in 12 cycles. The average conveyoring speed was 0.112 ± 0.00027 mm/s. The results in Figure 7B show that there is little difference between the 6:5 and 4:3 designs. We also increased the original actuation speed (Fig. 6) by a factor of 2, 4, and 8. The conveyoring velocity increased from the original 0.11 mm/s to 0.19 and 0.28 mm/s, and then decreased to 0.15 mm/s, as shown in Supplementary Figure S2C.

### Control strategies and displacements of soft ElectroSkin crawler robots

**FIG. 8.**
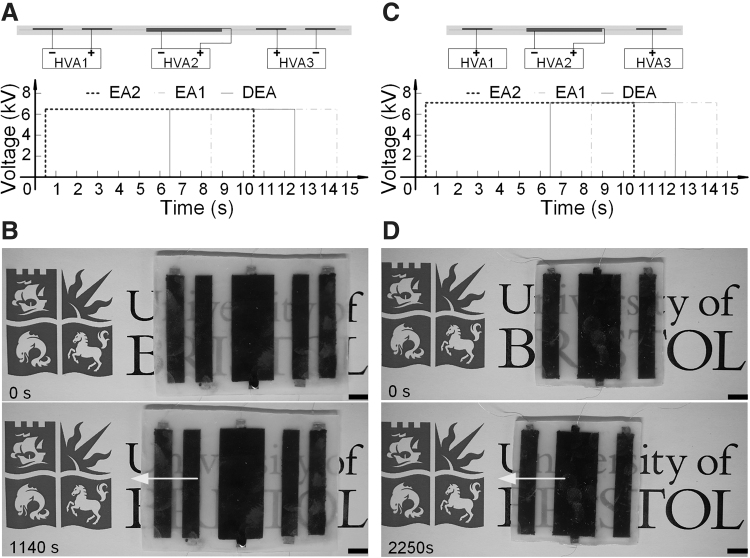
Voltage control strategy and displacement of the 6:5 and 4:3 soft ElectroSkin crawler robots. **(A)** Voltage control for the 6:5 ElectroSkin robot. **(B)** Displacement of the 6:5 ElectroSkin robot in 76 cycles (Supplementary Movie S4). **(C)** Voltage control for the 4:3 ElectroSkin robot. **(D)** Displacement of the 4:3 ElectroSkin robot in 150 cycles (Supplementary Movie S5). Scale bars denote 1 cm. Arrows denote the movement directions of the robotic crawlers.

Both the 6:5 and 4:3 ElectroSkin conveyor belt designs can be used to fabricate soft ElectroSkin crawlers. We encapsulated the ElectroSkin 6:5 and 4:3 conveyor belts with a 1 mm thick layer of Ecoflex 00–30 on both sides and removed the rigid frames. For the 6:5 crawler, presented in Figure 8A, 6.5 kV was applied to both the EA feet and DEA, whereas for the 4:3 crawler, presented in Figure 8C, 7kV was used. The 6:5 ElectroSkin crawler moved 6.0 mm in 76 cycles (0.079 mm per movement cycle; Fig. 8B). The 4:3 ElectroSkin crawler moved 10.2 mm in 150 cycles (0.068 mm per movement cycle; Fig. 8D). The crawling speeds of the 6:5 and 4:3 ElectroSkin crawlers were 0.005 and 0.0043 mm/s, respectively. Improved ElectroSkin designs using thinner materials with better dielectric and electrical properties will be investigated in the future to enhance the crawling speed.

## Discussions

The soft ElectroSkin conveyor can be further simplified as a 4:2 (four-electrode, two-unit) design, as presented in Figure 5C. In this design, an asymmetrical voltage control strategy was used for the two DEAs, each charged with opposite polarity such that an EA adhesive force is generated between them (Supplementary Fig. S3B). This design requires only two HVAs and exploits the difference in time constants between the EA (coplanar) capacitor and the DEA (parallel) capacitor (Supplementary Movie S3). We can also simplify the control in larger conveyor devices. Two HVAs, instead of three shown in Figure 2, can be used to control the 6:5 and 4:3 ElectroSkin conveyor belts, as presented in Supplementary Figure S6.

The 4:2 configuration can also be used to make an entirely soft DEA-EA robot crawler (Supplementary Movie S6). Soft ElectroSkin crawlers using more DEA units, such as a 6:3 (six-electrode, three DEA units) design, have also been developed and tested (Supplementary Movie S7). We attribute the slight turning to the slightly uneven encapsulation layers. The current ElectroSkins are only able to move in one dimension. Two-dimensional locomotion can be achieved by a 3 × 3 (or larger) DEA array. The ElectroSkin designs can be readily scaled up or down to match a specific soft robotic application, such as precise micropositioning of small objects or larger scale locomotion in robot exploration and rescue applications.

It is impractical to conduct both the theoretical and simulation modeling of dynamic DEA-EA movements and dynamic DEA and EA interactions as there is a lack of fundamental theories on high-voltage dynamic polarization of elastomeric materials yet and it is challenging to include environmental and surface texture factors into the model. We assume different electrode arrangements (such as the 6:5, 4:3, and 4:2) and voltage application strategies will bring different potential and electric field distributions, and DEA and EA interactions. To demonstrate an initial understanding of the potential interaction between DEA and EA, a static 2D electrostatic simulation of the 6:5 design was conducted and its electric field intensity and potential field distributions under two different voltage application strategies are shown in Supplementary Figure S4. It is clear from Supplementary Figures S4 and S5 (which shows the field intensity along the robot–substrate interfaces) that different DEA and EA interactions were produced by different voltage application strategies. This resulted in different conveyoring velocities of the same 6:5 design shown in Figure 7.

Both DEAs and soft EAs are made of compliant electrode and dielectric materials. Also, they both require a high voltage to energize them to produce desired behaviors. In contrast to soft parallel plate capacitors embodied in DEAs, most soft EAs are variable coplanar capacitors. It is desirable to have a high-permittivity dielectric sandwiched between DEAs or enclosing the electrodes of soft EAs. Dielectric breakdown of high-permittivity dielectrics usually follows closely a $\frac{1}{\sqrt{\varepsilon_{r}}}$ dependence.^41^ There is, therefore, a trade-off for dielectric material permittivity selection for both DEAs and soft EAs to achieve the largest DEA and EA forces while preventing dielectric breakdown. Furthermore, there is a trade-off for the stiffness selection for the dielectric encapsulation as the encapsulation should not be that stiff to limit the DEA strain on one hand and should be compliant enough to permit DEA strain but should be stiff enough to maintain sufficient prestretch of the dielectric membrane.

## Conclusions and Future Work

Soft active artificial skins are a critical missing component in robotics, wearable technologies, and health care. They are needed for skin-like coatings for robots and machines, enhancing sensing, manipulation, protection, and safety. Until now smart skins have predominantly been passive and any mechanical action has required external actuation, for example, using motors and tendons. In contrast, here we have shown ElectroSkin, a new class of skin-like composite structure that has the intrinsic capabilities of movement, locomotion, and active gripping, leveraging the benefits of DEA and EA actuations. In addition to realizing smart skin-like structures for conventional robots and delivering a new wearable technology, ElectroSkins can be configured into a wide range of thin-and-light active structures, and can be used to fabricate complete robots such as the soft ElectroSkin conveyor belts and pocketable crawler robots with different designs and control strategies we have presented in this article. These soft-smart composite devices demonstrate the effectiveness and potential of this active artificial skin to impact deployable and rescue robotics and industrial applications including pocketable robots, active grippers, and soft conveyor belts.

The contributions of this study include the design and development of ElectroSkin, an electroactive, entirely soft, and skin-like composite material and structure wherein actuation and adhesion are monolithically integrated. We leveraged this pocketable ElectroSkin material and structure to build robots capable of self-locomotion and soft conveyoring systems capable of moving objects using a range of electrode configurations and control logics to exploit combined DE actuation and EA adhesion and to minimize electrical supply channels. In addition, we conducted an empirical geometric optimization of two-electrode electroadhesion actuators by parametric exploration, fabrication, and testing.

DEA and EA both have low energy consumption characteristics; untethered and portable DEA or EA or DEA-EA devices are thus feasible.^36,42^ Based on the HVA number reduction strategy already described, we have also developed an untethered and portable 4:3 ElectroSkin conveyor (see Supplementary Fig. S7 and Supplementary Movie 8) based on a miniature microprocessor, a Li-Po battery, and two small and lightweight HVAs (see details in section 4 in the Supplementary Data). Triboelectric nanogenerators have been used to drive and produce self-powered DEAs^43^ and EAs.^44^ One possible solution to an all-soft untethered ElectroSkin crawler is to combine a further optimized ElectroSkin design with stretchable triboelectric nanogenerators,^45^ which will be investigated in the future. In addition, to better understand the interplay of fundamental actuation and adhesion mechanisms, we plan to conduct dynamic DEA-EA interaction modeling, considering the complex polarization and depolarization of elastomeric materials, surface texture, environmental conditions, and dynamic dielectric properties under varying high-voltage electric fields. This DEA-EA model may help estimate/predict conveyoring and crawling performances and inform better actuation/adhesion strategies.

## Supplementary Material

Supplemental data

Supplemental data

Supplemental data

Supplemental data

Supplemental data

Supplemental data

Supplemental data

Supplemental data

Supplemental data
