# Supplementary material for: All-Soft Skin-Like Structures for Robotic Locomotion and Transportation
Source: Soft Robot. 2020 Jun 2;7(3):309–20. doi: 10.1089/soro.2019.0059 (PMC7301317; doi:10.1089/soro.2019.0059)
Supplement: Supplemental data [file Supp_Data.pdf]

Supplementary Data

Section 1: Dielectric Elastomer Actuator and Electroadhesive Electrode Properties

We measured the dynamic electrode area change of our dielectric elastomer actuator (DEA), as presented in Supplementary Figure S1. In total, 5 kV was applied to the DEA, with a charging time of 15 s and a total cycle of 21 s. The DEA area strain was 31.8%. The measured actuation voltages and current draws of the 6:5 ElectroSkin conveyor design are shown in Supplementary Figure S2A and B, respectively. We also increased the original actuation speed (Fig. 6) by a factor of 2, 4, and 8. The conveyoring velocity increased from the original 0.11 mm/s to 0.19 and 0.28 mm/s, and then decreased to 0.15 mm/s, as shown in Supplementary Figure S2C. The resistor–capacitor (RC) time constants of the DEA and electroadhesive (EA) units were all <0.05 s. However, it takes time for the DEA unit to achieve the maximum strain and the EA unit to produce enough force for transporting materials.

Section 2: ElectroSkin 4:3 and 4:2 Design Operation Principles

In this study, only the 4:3 and 4:2 ElectroSkin conveyor operation principles are shown (Supplementary Fig. S3) as the 4:3 and 4:2 ElectroSkin crawler operation principles are similar (as presented in Fig. 2). The same voltage control strategy 1 shown in Figure 7A was used for the 4:3 conveyor belt (Supplementary Movie S2) and crawler (Supplementary Movie S5), whereas an asymmetrical voltage control strategy was used for the two DEAs for the 4:2 conveyor belt (Supplementary Movie S3) and crawler (Supplementary Movie S6).

The ElectroSkin 4:3 operation procedure contains six steps: (i) complete the necessary electrical connections and put the object on the conveyor belt when no voltage is applied; (ii) turn on the EA2 electrode and the object is gripped due to EA force; (iii) turn on the DEA and the object can be moved to the right due to simultaneous dielectric elastomer (DE) actuation and EA adhesion; (iv) turn off the EA2 and turn on the EA1 so that the other end of the object is gripped; (v) turn off the DEA and the object can be moved to the right again due to DE strain release and EA adhesion; (vi) turn off the EA1 and start the next cycle if needed. After this cycle, as shown in Supplementary Figure S3A, the object has moved laterally a distance of d_3. For moving objects to the left, EA1 electrode should be turned on first rather than EA2 electrode.

The ElectroSkin 4:2 operation procedure contains four steps: (i) complete the necessary electrical connections and put the object on the conveyor belt when no voltage is applied; (ii) turn on the DEA2 and the object is gripped due to EA force and can be moved to the left due to DE actuation; (iii) turn on the DEA1 using a smaller voltage and the object is being moved forward slightly due to simultaneous DE actuation and EA adhesion; (iv) turn off the DEA1 and DEA2, and start the next cycle if needed. After this cycle, as shown in Supplementary Figure S3B, the object has moved laterally a distance of d_4. For moving objects to the right, DEA1 should be turned on first.

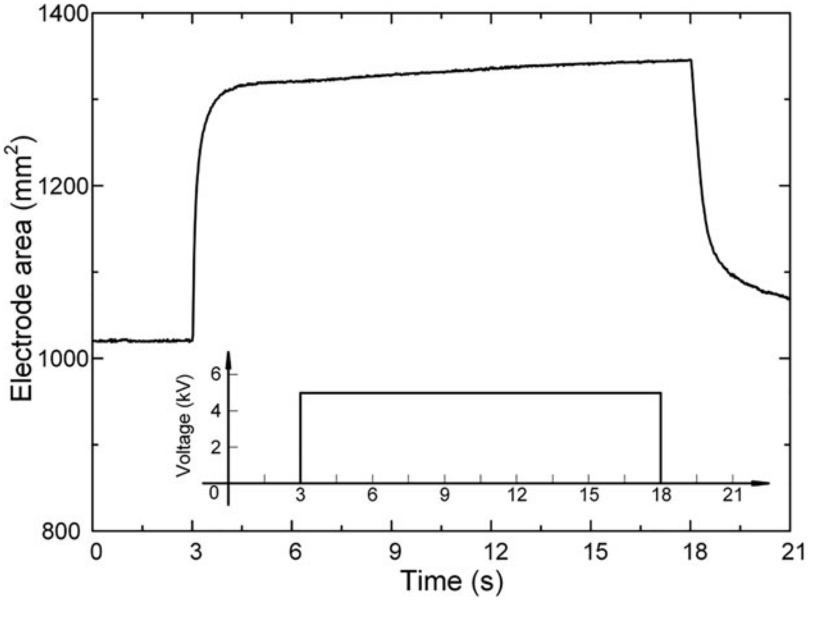

SUPPLEMENTARY FIG. S1. Dielectric elastomer actuator dynamic electrode area change under 5 kV with a charging time of 15 s and a total cycle of 21 s.

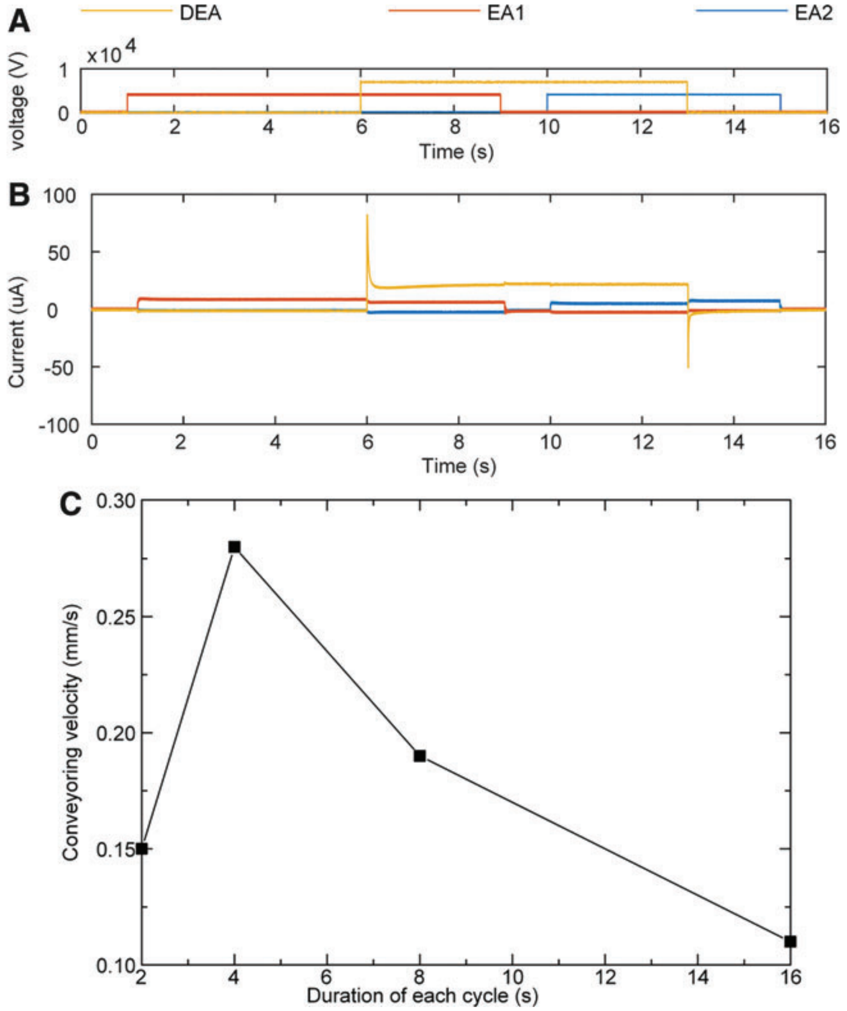

SUPPLEMENTARY FIG. S2. 6:5 ElectroSkin actuation voltage, current draw, and conveyoring velocity against different cycle durations. **(A)** The measured actuation voltages of the 6:5 ElectroSkin conveyor. **(B)** The measured currents of the 6:5 ElectroSkin conveyor. **(C)** Conveyoring velocities under different cycle durations. DEA, dielectric elastomer actuator; EA, electroadhesive.

The RC time constants of the EA and DEA units were <0.05 s as already mentioned. The DEA mechanical relaxation time was ∼1.5 s. The EA physical dielectric relaxation time when gripping papers was <0.1 s, much quicker than the DEA mechanical response. The working operation of 4:2 ElectroSkin designs was based on this response difference. The EA dielectric relaxation time when gripping plastics can be in the order of tens of seconds or more, which will necessitate an actuation strategy different to that already described for moving papers. This will be investigated in the future.

Section 3: Two-Dimensional Electrostatic Simulation of the ElectroSkin Designs

A static two-dimensional electrostatic finite element analysis (FEA) simulation, using the Finite Element Method Magnetics (FEMM) software, was conducted to manifest the interactions between DEA and EA under different voltage application strategies. Different electrode arrangements and voltage application strategies will bring different potential and electric field distributions, and DEA and EA interactions. We selected two different voltage application strategies of the encapsulated 6:5 ElectroSkin design, as shown in Supplementary Figure S4. The EA interaction between the bottom DEA electrode (d) and the two EA electrodes near (b and e) it is different from Supplementary Figure S4A to B. There is a slight difference in the magnitude of field density along the crawling surface substrate of the encapsulated 6:5 ElectroSkin design when applying different voltage strategies, as shown in Supplementary Figure S5. There is a stronger EA interaction in the voltage application strategy shown in Supplementary Figure S4B as there is a clear potential difference between the electrode d and the electrodes b and e.

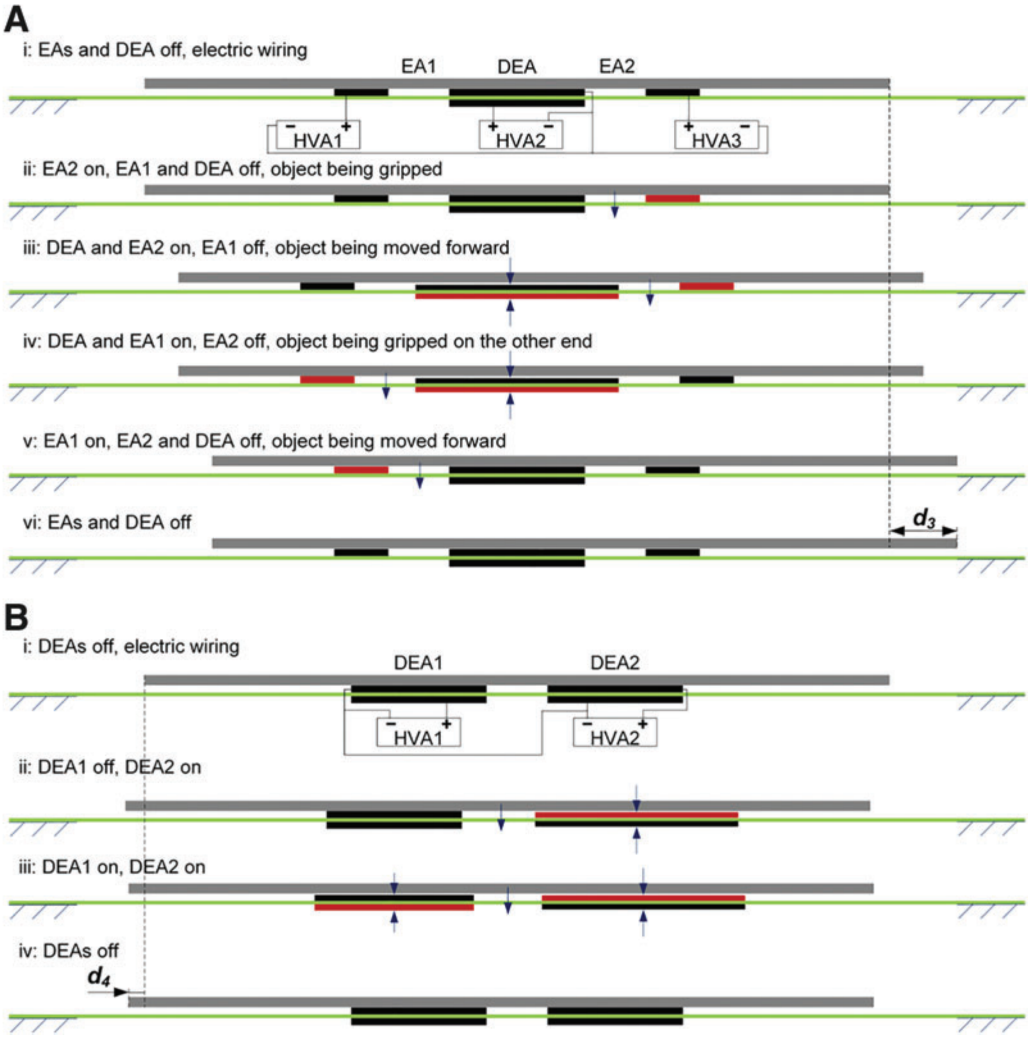

SUPPLEMENTARY FIG. S3. ElectroSkin 4:3 and 4:2 design operation principles. **(A)** ElectroSkin 4:3 design operation principles. **(B)** ElectroSkin 4:2 design operation principles. HVA, high-voltage amplifier.

Section 4: ElectroSkin Designs HVA Number Reduction and Development of a Portable 4:3 ElectroSkin Conveyor

Two HVAs, instead of three, can be used to control the 6:5 and 4:3 ElectroSkin conveyor belts, as presented in Supplementary Figure S6. For both the 6:5 and 4:3 ElectroSkin conveyor belt design and based on the electric connections shown in Supplementary Figure S6, turning on the HVA1 moves an object backward. Turning on the HVA2 then grips the object. Turning off the HVA1 but maintaining the HVA2 on moves the object further backward. Moving forward requires the opposite electrical connections.

Based on the design strategy shown in Supplementary Figure S6B, we developed an untethered and portable 4:3 ElectroSkin conveyor based on a miniature Arduino Beetle microprocessor (DFRobot, China), a Li-Po battery (Dragonmarts, China), and two small and lightweight EMCO Q50 HVAs (XP Power, Singapore). The design layout and its three-dimensional CAD model are shown in Supplementary Figure S7A and B, respectively. The prototype is shown in Supplementary Figure S7C and the demonstration of its movements can be seen in Supplementary Movie S8. A high-voltage bleed resistor connected in series to the HVAs is suggested to stabilize the current output if there is a spike for safety considerations and for discharging uses.

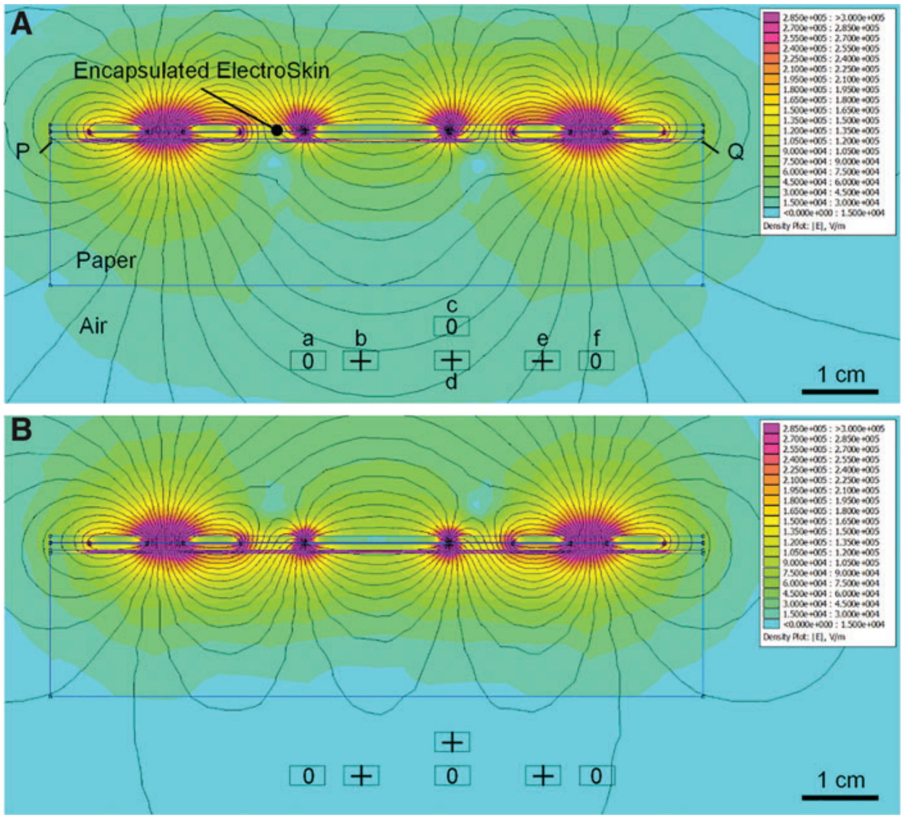

SUPPLEMENTARY FIG. S4. Static electric intensity (V/m) and equipotential (V) distribution simulation results of the encapsulated 6:5 ElectroSkin design. **(A)** Three kilovolts was applied to electrodes b, d, and e and 0 V was applied to electrodes a, c, and f. **(B)** Three kilovolts was applied to electrodes b, c, and e and 0 V was applied to electrodes a, d, and f. ‘‘+’’ denotes 3 kV. ‘‘0’’ denotes the ground. Default materials from the FEMM material library were used, including the air (dielectric constant of 1) and paper (dielectric constant of 2.5). Customized materials including silicone (dielectric constant of 2.8) and VHB (dielectric constant of 4) were designated and defined into the specific areas in the encapsulation ElectroSkin. FEMM, Finite Element Method Magnetics; VHB, very high bond.

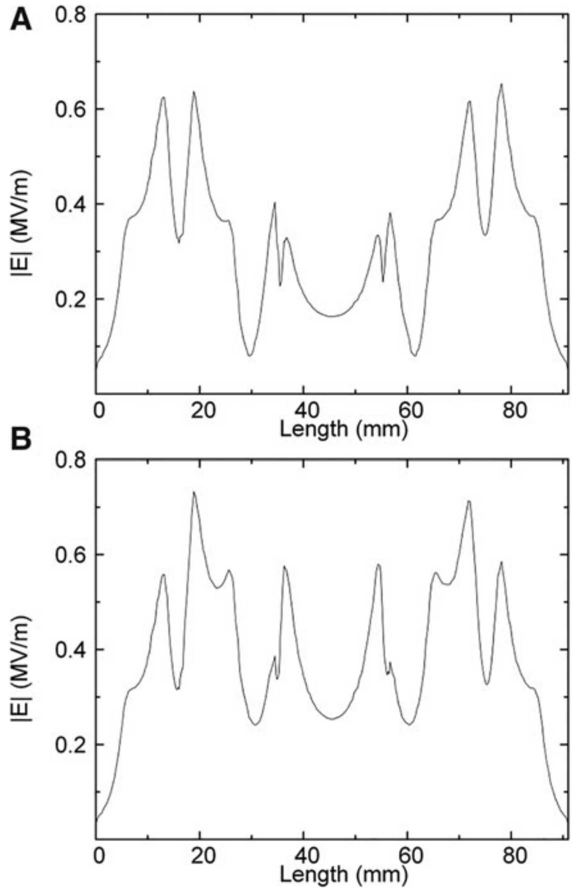

SUPPLEMENTARY FIG. S5. Magnitude of field density, | E |, along the line PQ (shown in Supplementary Fig. S4A, the surface of the paper substrate) of the encapsulated 6:5 ElectroSkin design shown in **(A)** Supplementary Fig. S4A and **(B)** Supplementary Fig. S4B.

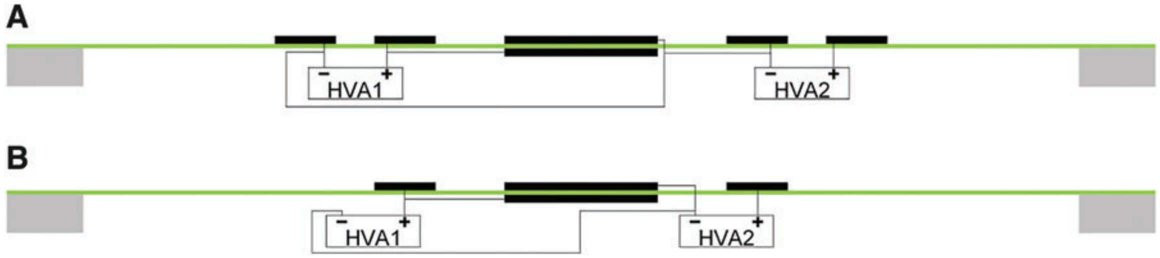

SUPPLEMENTARY FIG. S6. HVA number reduction strategies for the ElectroSkin designs. **(A)** One HVA number reduction strategy for the 6:5 ElectroSkin conveyor belt design. **(B)** One HVA number reduction strategy for the 4:3 ElectroSkin conveyor belt design.

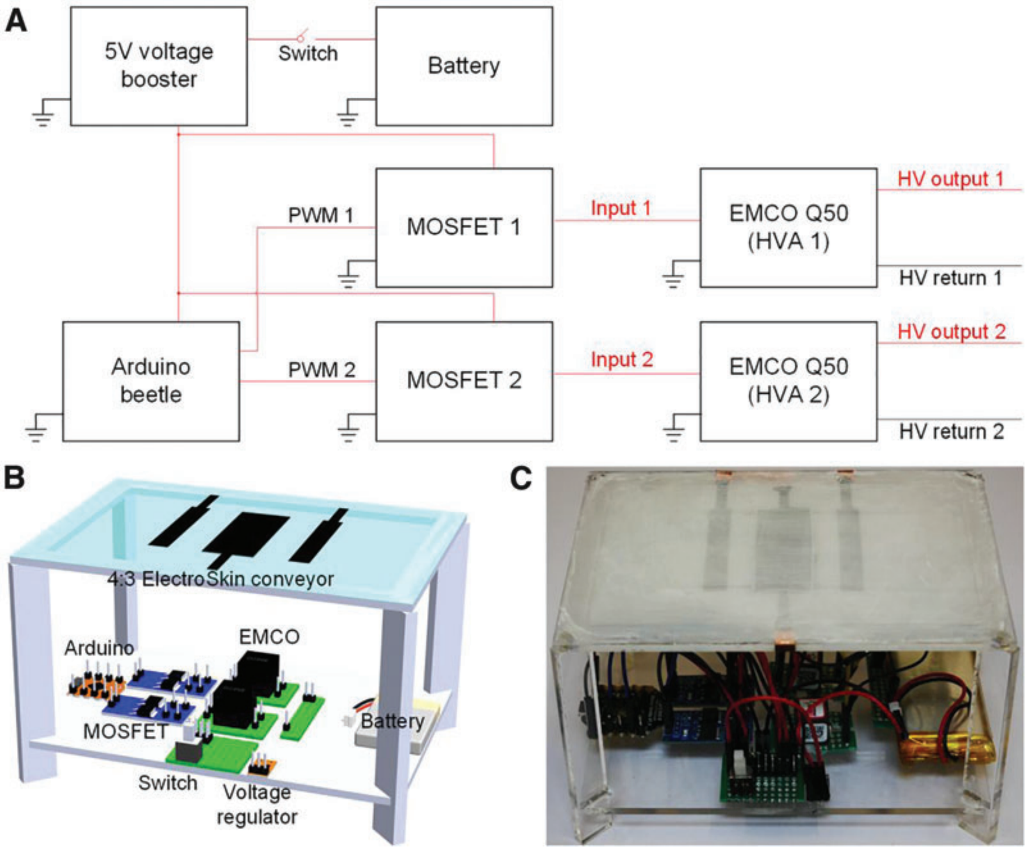

SUPPLEMENTARY FIG. S7. Development of an untethered and portable 4:3 ElectroSkin conveyor. **(A)** Schematic diagram of the untethered 4:3 ElectroSkin conveyor design. **(B)** Three-dimensional CAD model of the untethered 4:3 ElectroSkin conveyor design. **(C)** Prototype of the untethered 4:3 ElectroSkin conveyor design.
